# Simulated space radiation-induced mutants in the mouse kidney display widespread genomic change

**DOI:** 10.1371/journal.pone.0180412

**Published:** 2017-07-06

**Authors:** Mitchell S. Turker, Dmytro Grygoryev, Michael Lasarev, Anna Ohlrich, Furaha A. Rwatambuga, Sorrel Johnson, Cristian Dan, Bradley Eckelmann, Gwen Hryciw, Jian-Hua Mao, Antoine M. Snijders, Stacey Gauny, Amy Kronenberg

**Affiliations:** 1 Oregon Institute of Occupational Health Sciences, Oregon Health & Science University, Portland, Oregon, United States of America; 2 Department of Molecular and Medical Genetics, Oregon Health & Science University, Portland, Oregon, United States of America; 3 Biological Systems and Engineering Division, Lawrence Berkeley National Laboratory, Berkeley, California, United States of America; Georgetown University, UNITED STATES

## Abstract

Exposure to a small number of high-energy heavy charged particles (HZE ions), as found in the deep space environment, could significantly affect astronaut health following prolonged periods of space travel if these ions induce mutations and related cancers. In this study, we used an *in vivo* mutagenesis assay to define the mutagenic effects of accelerated ^56^Fe ions (1 GeV/amu, 151 keV/μm) in the mouse kidney epithelium exposed to doses ranging from 0.25 to 2.0 Gy. These doses represent fluences ranging from 1 to 8 particle traversals per cell nucleus. The *Aprt* locus, located on chromosome 8, was used to select induced and spontaneous mutants. To fully define the mutagenic effects, we used multiple endpoints including mutant frequencies, mutation spectrum for chromosome 8, translocations involving chromosome 8, and mutations affecting non-selected chromosomes. The results demonstrate mutagenic effects that often affect multiple chromosomes for all Fe ion doses tested. For comparison with the most abundant sparsely ionizing particle found in space, we also examined the mutagenic effects of high-energy protons (1 GeV, 0.24 keV/μm) at 0.5 and 1.0 Gy. Similar doses of protons were not as mutagenic as Fe ions for many assays, though genomic effects were detected in *Aprt* mutants at these doses. Considered as a whole, the data demonstrate that Fe ions are highly mutagenic at the low doses and fluences of relevance to human spaceflight, and that cells with considerable genomic mutations are readily induced by these exposures and persist in the kidney epithelium. The level of genomic change produced by low fluence exposure to heavy ions is reminiscent of the extensive rearrangements seen in tumor genomes suggesting a potential initiation step in radiation carcinogenesis.

## Introduction

The risk of cancer from exposure to charged particles that induce mutations is a long-term concern for astronauts on prolonged missions in deep space [1–4]. The most common charged particles in space are sparsely ionizing protons present in the trapped radiation belts, in solar particle events (SPE), and among the galactic cosmic rays (GCR) [1]. However, charged particles with high linear energy transfer (LET) that are present in the GCR are of substantial concern for the induction of mutations due to their ability to deposit more energy locally generating the potential for even a single particle traversing a cell nucleus to cause complex DNA damage and resultant mutations.

Most mutagenesis studies with charged particles use cell culture systems [5–11] and/or mouse models [12–18] to identify mutagenic effects. The main advantages of cell culture systems are reduced time and cost for the complete assay because cultured cells are actively dividing. Human cells can also be studied in culture, which adds relevance to those studies. In contrast, rodent models allow mutation formation to occur in the normal tissue environment [19]. For many normal tissues, only a small fraction of cells are in cycle at any snapshot in time (i.e., a few percent). As a consequence, *in vivo* models are useful surrogates for the mutagenic effects of charged particles that will occur in the tissues of astronauts on space missions.

We use an *in vivo* system to study charged particle mutagenesis in the intact kidney epithelium in which the selectable autosomal mouse *Aprt* gene, located on chromosome 8, detects the full spectrum of mutations, from small intragenic changes to whole chromosome loss [12–15, 19]. Charged particle exposure induces *Aprt* mutant cells, which persist for extended times in the kidney [12, 13] until isolated from the dispersed kidney via selection of the primary cells in culture. Selection in culture allows the mutant clones that formed in the body to be expanded for a comprehensive analysis of charged particle mutagenesis using a variety of endpoints.

Here we used our *in vivo* system to examine mutant frequencies, mutation spectrum at the target chromosome (chromosome 8), translocations affecting chromosome 8, and mutations affecting the rest of the genome in *Aprt* mutant cells to create a comprehensive picture of ^56^Fe ion mutagenesis in the intact kidney epithelium at doses ranging from 0.25 to 2.0 Gy. These doses provide a fluence range of 1 to 8 particle traversals per cell nucleus. For comparison with a sparsely ionizing, low LET charged particle, we also examined proton mutagenesis at 0.5 and 1.0 Gy. These proton doses are also relevant for large solar particle events, which occur sporadically and require very large numbers of proton traversals per cell nucleus, in contrast to the small numbers of densely ionizing Fe ions. The results of this study show that a low fluence of high LET Fe ions leads to extensive genomic mutations in a subset of exposed kidney cells, which we identified by selection for mutations affecting *Aprt* expression.

## Materials and methods

### Mouse strain and irradiations

All animal work was conducted under protocols approved by Oregon Health and Science University and Brookhaven National Laboratory. The experimental mice were *Aprt* heterozygous (*Aprt*^*+/-*^) B6D2F1 hybrids from C57BL/6 X DBA/2 crosses. These F1 hybrid mice were created anew for each round of irradiations (see below) to ensure that the mice were genetically identical for all experiments. The C57BL/6 parent carries the *Aprt* knockout allele and has been backcrossed and maintained in this strain background in the Turker laboratory for >20years. To maintain the C57BL/6 *Aprt*^*+/-*^ strain, they are bred biannually to C57BL/6 wild type mice obtained from Jax Labs (Bar Harbor, Maine). The DBA/2 parents carry the wild type *Aprt* allele and these mice are obtained for each round of breedings from Jax Labs. To produce the experimental B6D2F1 *Aprt*^*+/-*^ mice, in most cases male C57BL/6 *Aprt*^*+/-*^ mice were bred with female DBA/2 mice. In some cases (~25%) the reverse breedings were performed, i.e., female C57BL/6 *Aprt*^*+/-*^ with male DBA/2 mice. Males and female B6D2F1 *Aprt*^*+/-*^ mice in approximately equal numbers were exposed to ^56^Fe ions (1 GeV/amu, 151 keV/μm; 0.25 Gy to 2.0 Gy) or protons (1 GeV, 0.24 keV/μm; 0.5 Gy to 4.0 Gy) at the NASA Space Radiation Laboratory at Brookhaven National Laboratory (BNL) at dose rates of 0.5 Gy/minute for doses 0.5 Gy or lower and 1 Gy/minute for doses at or higher than 1 Gy. The estimated fluence for Fe ions was 1, 2, 4, and 8 particle traversals per cell nucleus for 0.25, 0.5, 1.0, and 2.0 Gy, respectively. These figures were calculated according to standard methods using the formula: fluence (per μm^2^) = 6.24 D/L, where D is the dose in Gy, and L is the LET in keV/μm. As an example, for a dose of 1 Gy, the fluence (per μm^2^) of 1 GeV/amu Fe ions would be 0.041; for the same dose of 1 GeV protons, the fluence (per μm^2^) would be 26. Comparing the number of protons to deliver 1 Gy to the number of Fe ions to deliver 1 Gy demonstrates that it takes approximately 630 times more protons to deliver the same dose as a single Fe ion [20, 21]. The age range of the mice of six to seven months of age was chosen to approximate a biological age similar to that of a mid-career astronaut.

Whole body exposures were conducted without anesthesia in small Plexiglas boxes with ventilation holes. Mice were positioned with their sides perpendicular to the beam path to minimize changes in energy deposited through the animal. For all sets of exposures, termed runs (see Table 1), 18 mice were used as sham controls and between 21 and 27 mice were exposed for a given dose. All animal work was conducted under protocols approved by OHSU and BNL.

**Table 1:** Mutant frequencies and cytotoxicity in charged particle exposed kidney epithelial cells.

Dose	Run^a^	N^b^	MF (X 10^−4^)^c^	Relative MF increase^d^	CE^e^
0	1	9	0.7 (0.4, 1.3)	----	1.00
2 Gy Fe	1	17	3.6 (2.5, 5.2)	4.9; **p<0.001**	0.57; **p<0.01**
4 Gy H	1	21	ND	2.8; p<0.001*	0.71; **p<0.01**
0	2	16	1.4 (0.9, 2.2)	----	1.00
0.5 Gy Fe	2	27	3.1 (2.3, 4.1)	2.1; **p = 0.004**	0.86; p = 0.078
0	3	17	2.3 (1.7, 3.1)	----	1.00
1.0 Gy Fe	3	18	5.8 (4.3, 7.9)	2.5; **p<0.001**	0.72; **p = 0.006**
0.25 Gy Fe	3	18	3.6 (2.6, 5.1)	1.6; **p = 0.034**	0.97; p>0.08
1.0 Gy H	3	19	3.2 (2.3, 4.5)	1.4; p = 0.109	0.87; p>0.08
0	4	14	1.3 (0.9, 2.1)	----	1.00
0.5 Gy H	4	21	1.8 (1.3, 2.6)	1.4; p = 0.279	0.82, p = 0.064

^a^ Each run represents a different shipment of mice to BNL and exposures to the doses shown.

^b^ Number of mice tested

^c^ MF represents *Aprt* mutant frequencies.

^d^ Relative mutant MF (mutant frequency) increase represent mutant frequencies for charged particle-exposed kidneys relative to spontaneous mutant frequencies for that run. Significant p values are bolded.

^e^ CE represents cloning efficiencies for epithelial cells removed from charged particle-exposed kidneys relative to unexposed kidneys. Significant p values are bolded.

* Mutant frequency data from Kronenberg et al, 2013 [13].

### Selection of Aprt mutants

To select mutants that arose after *in vivo* exposures, kidneys that were apparently normal based on size and color were harvested at 3–4 months post exposure from mice killed by exposure to CO_2_ at a fill rate of 20% using procedures required by the OHSU IACUC. Primary cells were isolated from enzymatically digested kidneys (usually one kidney per mouse), as described elsewhere [22, 23], and ten aliquots representing 95% of each kidney were each seeded in 100 mm^2^ dishes in the presence of 2’6-diaminopurine (DAP) (Sigma, St. Louis, Missouri) to select *Aprt* mutant cells [23, 24]. The remaining cell suspensions were used to determine the number of cells capable of producing primary clones per kidney in the absence of selection. On average, a kidney from a non-irradiated mouse yielded approximately 20,000–30,000 primary clones. The *Aprt* mutant clones were identified 5–6 weeks after initiation of DAP selection. Mutant clones were expanded in DMEM (Gibco/Life Technologies, Grand Island, New York) supplemented with 15% fetal bovine serum until confluent in a T25 cm^2^ flask, when DNA was isolated for molecular analysis. A limited number of non-mutant (i.e., non-selected) clones were also expanded to serve as controls. Each clone was plated in one well of a 24 well plate and when confluent the cells were split into three wells. Finally, when the three wells were confluent, the cells were seeded into one T25 cm^2^ flask and split 1:2 thereafter for DNA preparations and cytogenetic analyses, which was performed as described elsewhere [14].

### LOH analyses

DNA preparations were isolated from expanded DAP resistant clones that were confluent in 25 cm^2^ flasks using a conventional “salting out” method [25]. Results from spontaneous mutants have been described previously [26, 27]. In most cases, only one kidney per mouse was used. No more than 5 mutants were examined from a given kidney, and in most cases 3 or fewer mutants were examined. To determine the mechanism underlying each *Aprt* mutation, each DNA preparation was examined for retention or loss of heterozygosity by PCR amplification of 13 polymorphic microsatellite loci on chromosome 8 (Invitrogen, Carlsbad, CA), including a microsatellite sequence located immediately upstream of *Aprt* [28]. To identify LOH events on additional chromosomes, analyses were performed for one polymorphic locus on each of 11 pairs of additional chromosomes (*D2Mit356*, *D3Mit29*, *D5Mit161*, *D6Mit243*, *D7Mit246*, *D9Mit191*, *D11Mit136*, *D12Mit136*, *D13Mit66*, *D14Mit165*, and *D15Mit159*)[29]. The LOH analyses were conducted at the Plant-Microbe Genomics Facility at Ohio State University, which uses an ABI Prism 3700 DNA analyser to separate fluorescently-labelled PCR products.

### SNP analysis for whole genome scan

Purified DNA samples (400 ng/sample) were sent to the vendor (Neogen/Gene Seek, Lincoln, NE) for whole genome SNP analysis using the GigaMUGA platform. Uninformative SNP’s for the B6D2F1 genotype were removed and the remaining SNP’s were assigned a numerical value (“A” allele = -1, “B” allele = -4, “Heterozygous” = +4, and “Unidentified” = 0). Regions of LOH were visualized for each pair of chromosomes using the ggplot package in “R” software, using default parameters. The regions of LOH were manually scored.

### Statistical analysis

Cytotoxicity for kidney epithelial cells exposed *in vivo* was analyzed as a function of dose. The effect of dose on cloning efficiency was examined using multiple linear regression applied to the log transformed number of clones according to standard methods [26]. For *in vivo* determinations of *Aprt* mutant fractions, the data were analyzed as a function of dose using logistic regression for binomial proportions with an allowance made for overdispersion according to standard methods [30]. The distribution of mutational events for different radiation doses or conditions was analyzed using Fisher’s exact test. To avoid biasing the mutation spectra analyses, we used all mutant cells collected (with the exception of not using more than 5 per kidney, as noted above). The analyses do not require the total number of mutant cells examined for each dose to be constant. Tests reported reflect the degree to which the distribution of proportions (i.e. relative frequencies) differ according to dose and these proportions are conditional upon whatever the total number of mutant cells might be observed.

Log-binomial regression, which yields estimates of probability rather than estimated odds, was used to analyze the proportion of affected loci and proportion of affected clones as a function of dose. This methodology was also applied to each of the 11 pairs of chromosomes to determine whether the proportion of events (relative to total affected loci or total number of clones) differed according to dose. Likelihood ratio chi-square statistics and corresponding p-values from these log-binomial models were used to score and evaluate the strength of association between dose and proportion of events; adjustment (Bonferroni correction) was made to account for multiple testing. Poisson regression was used to model the association between dose and the mean number of affected loci per affected clone.

## Results

### Aprt mutant frequencies and cytotoxicity as a function of dose

**Fig 1:**
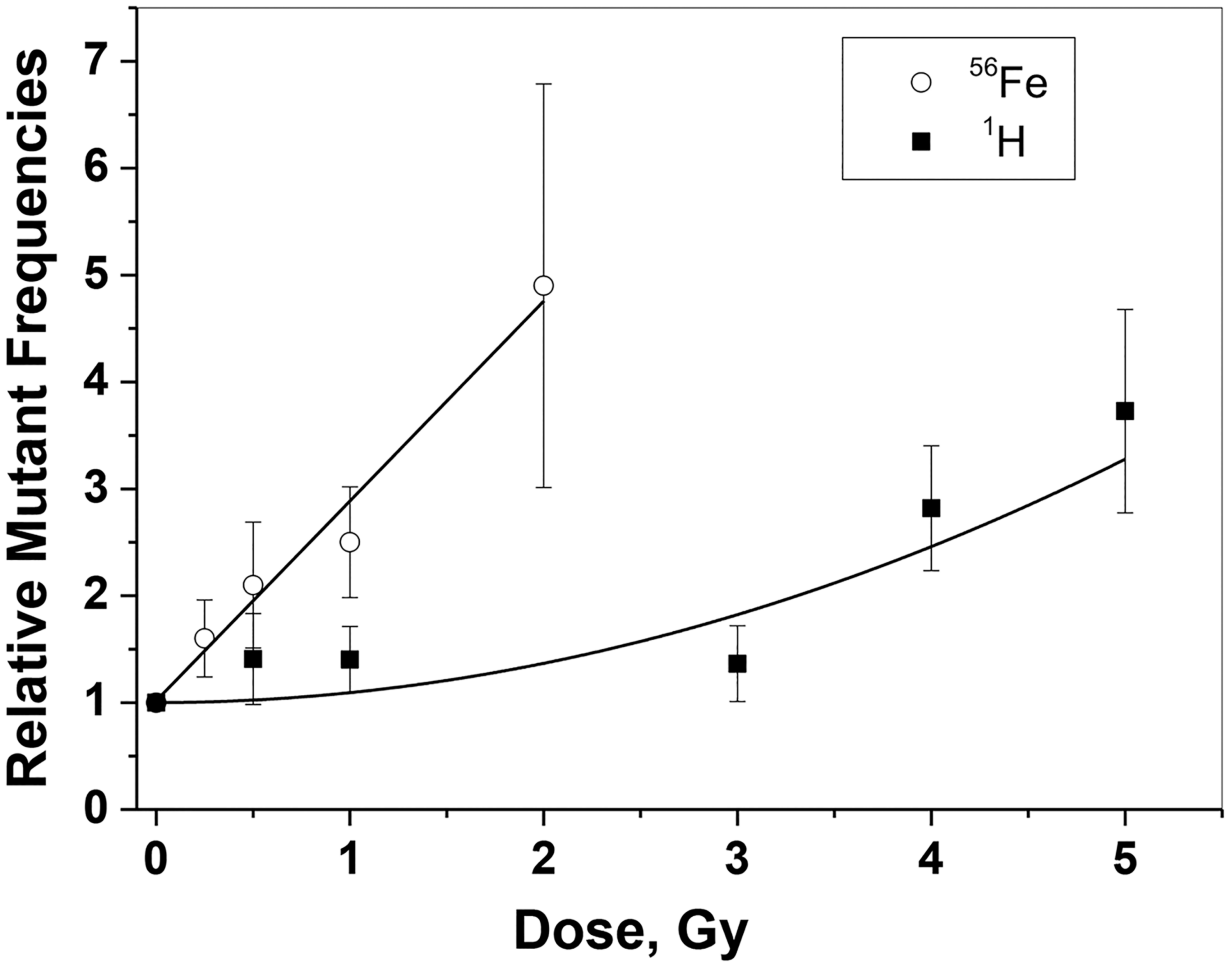
*Aprt* mutant frequencies. *Aprt* mutant frequencies were determined for Fe ions and protons and plotted relative to spontaneous mutant frequencies. Data for 3–5 Gy protons was taken from a prior study [13]. See text for more details.

The effects of Fe ion or proton exposures on *Aprt* mutant frequency and toxicity in the kidney epithelium were measured at four separate times (termed “runs”). Each run reflects a separate shipment of mice to BNL for exposure. For the first run, we measured the effects of 2 Gy Fe ions or 4 Gy protons (toxicity only for protons); for the second run we measured the effects of a dose of 0.5 Gy Fe ions; for the third run we measured the effects of 1.0 or 0.25 Gy Fe ions, or of 1.0 Gy protons; and for the fourth run we measured the effects of 0.5 Gy protons. For each run, a non-exposed control group of mice was used for comparison. Results for mutant frequencies and toxicity are presented in Table 1 and Figs 1 (mutant frequencies) and 2 (toxicity). The Table and Figures are supplemented with data from a prior study using doses of 3, 4, or 5 Gy protons [13].

**Fig 2:**
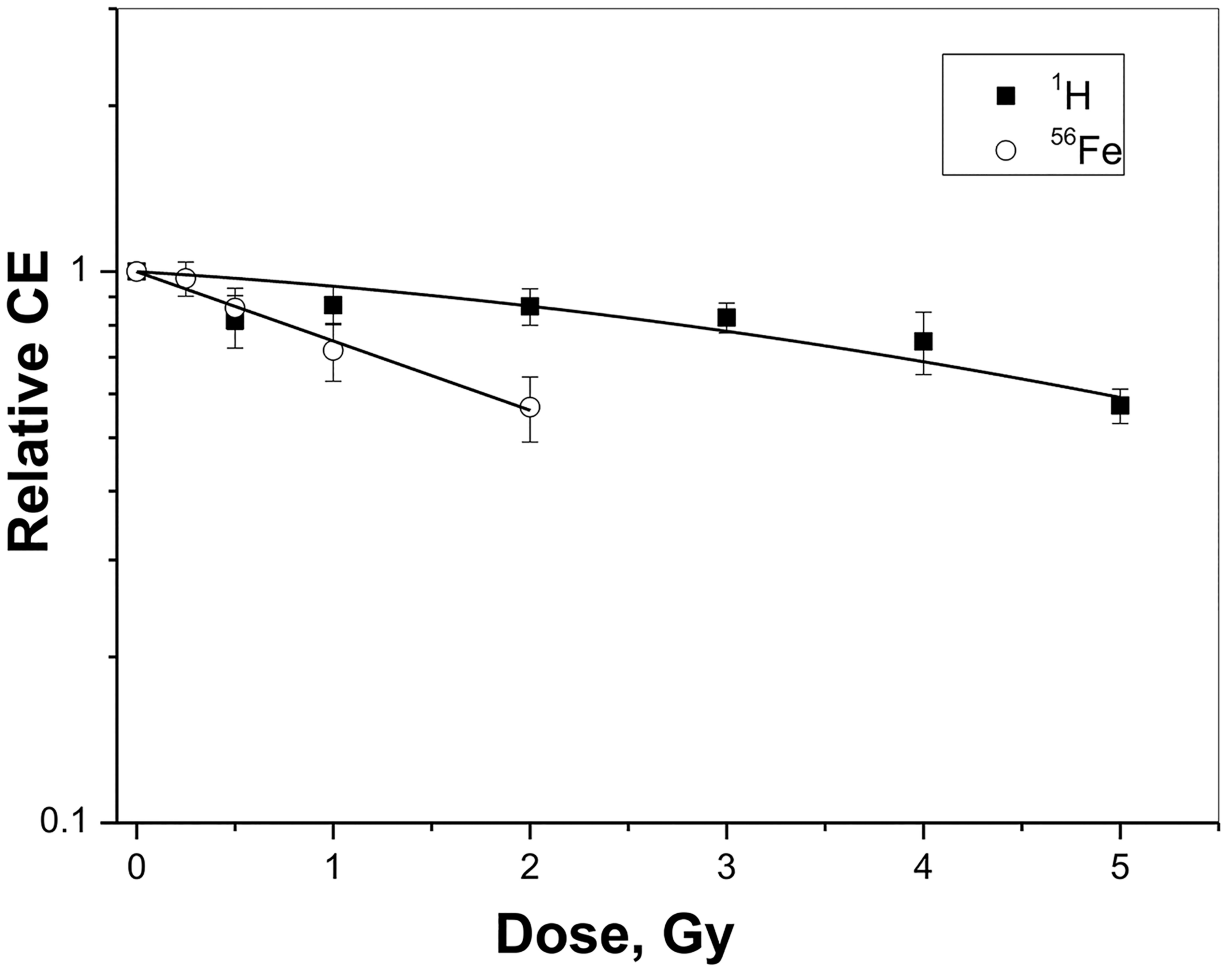
Cytotoxicity for cloning efficiencies for kidney epithelial cells removed from charged particle-exposed kidneys. Cloning efficiencies (CE) were determined for kidneys exposed to Fe ions and protons and plotted relative to cloning efficiencies for untreated kidneys. Data for 3–5 Gy protons were taken from a prior study [13].

Statistically significant increases for mutant frequencies were observed at all doses of ^56^Fe ions: 0.25 Gy (p = .034), 0.5 Gy (p = .004), 1.0 Gy (p < .001), and 2.0 Gy (p < .001). Because the spontaneous mutant frequencies ranged from 0.7 to 2.3 X 10^−4^ in the individual runs, we plotted mutant frequencies for exposed kidneys relative to controls for that run (Fig 1). The plot shows a linear increase in mutant frequencies as a function of dose with an adjusted R-squared value of 0.97. In contrast, although increases in *Aprt* mutant frequencies were observed at 0.5 and 1.0 Gy protons (~1.4 fold in both cases), these increases were not statistically significant (p = 0.279 and 0.109, respectively) (Table 1). Adding these data to mutant frequency data obtained at higher doses in a prior study (3.0, 4.0, and 5.0 Gy) [13] confirmed that the proton dose response is curvilinear, with little or no change at the lower doses and a doubling dose estimated at approximately 2–2.5 Gy (Fig 1). The adjusted R-squared value for all proton mutant frequencies is 0.93.

Cloning efficiencies for the harvested kidney epithelial cells were determined at 3–5 months after exposure to measure residual toxicity. For ^56^Fe ions, significant reductions were observed at the two highest doses, 1.0 (p = .006) and 2.0 Gy (p < .01). The adjusted R-squared value for all four values was 0.98 and fit a linear curve (Fig 2). The adjusted R-squared value for all five doses of protons was 0.81 and fit a linear quadratic curve suggesting a shoulder at the lower doses.

### Chromosome 8 mutation spectra for ^56^Fe ions and protons in the mouse kidneys

**Fig 3:**
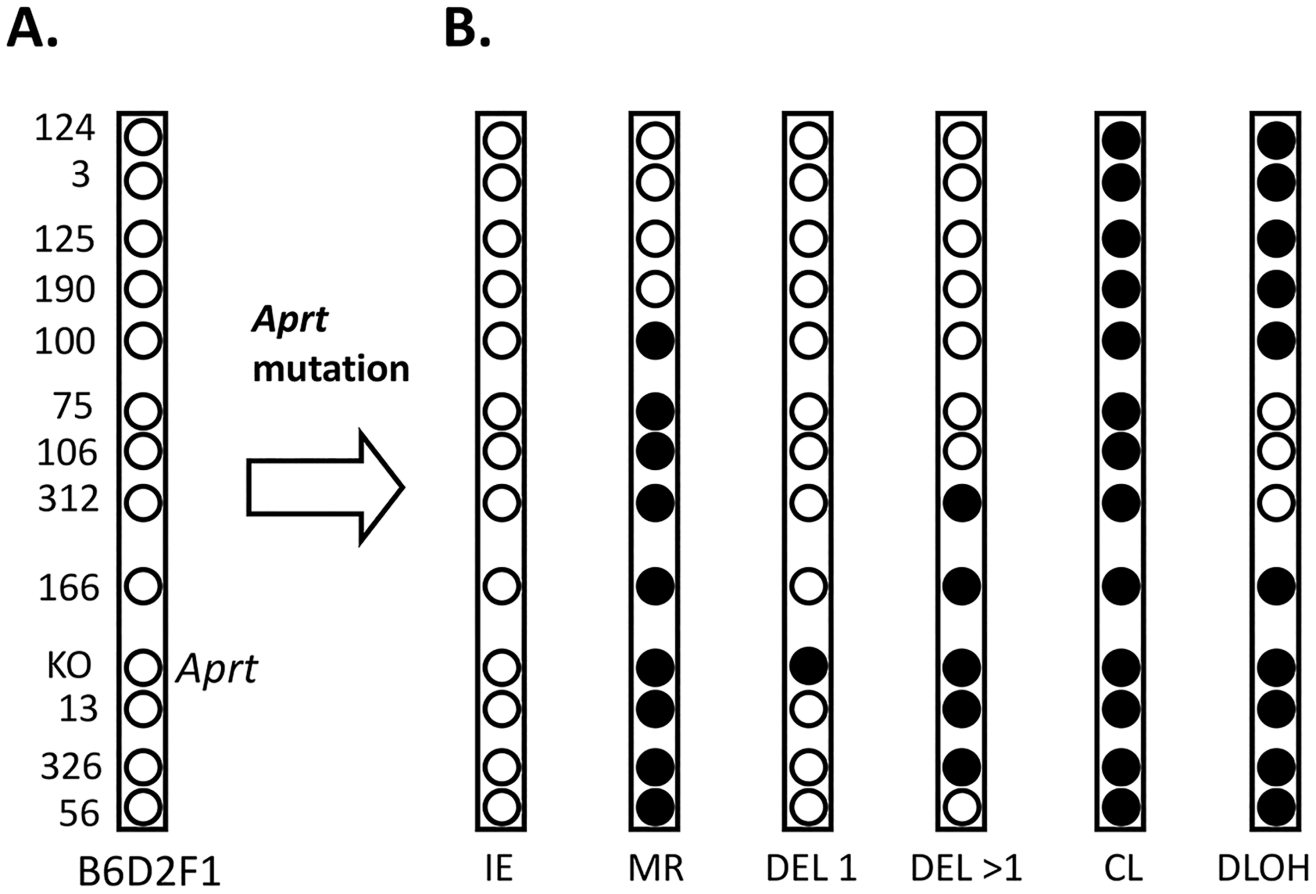
Loss of heterozygosity (LOH) patterns reveal different mutational events. **A.** The relative locations of polymorphic loci on mouse chromosome 8 that differ between the C57BL/6 and DBA2 mouse strains and were used to deduce the specific mutation present in each *Aprt* mutant examined in this study. The B6 (C57BL/6) derived chromosome contains a knockout (KO) *Aprt* allele that is non-functional. The D2 (DBA/2) derived chromosome contains an expressed, wild type *Aprt* allele, which is the target of DAP selection (i.e. loss of expression of this *Aprt* allele due to a mutational event allows a cell to grow in the presence of DAP in the culture medium). The numbers on the left side identify the chromosome 8 microsatellite loci that were examined in this study. These numbers omit the D8Mit prefix. **B.** The PCR-based molecular analysis for loss (closed circles) or retention (open circles) of heterozygosity for polymorphic microsatellite sequences on mouse chromosome 8 in the *Aprt* mutant cells yields LOH patterns that can be used to classify each mutational event into one of 8 different categories. These are intragenic events (IE), apparent mitotic recombination (MR), interstitial deletion of *Aprt* polymorphic locus only (DEL 1), multilocus deletion (DEL > 1), chromosome loss (CL), and discontinuous LOH (DLOH). See text for more details.

The expressed *Aprt* allele in the B6D2F1 heterozygous mice is derived from the DBA/2 parent. Thus, large mutational events leading to loss of *Aprt* expression result in LOH events for loci on the DBA/2-derived chromosome 8 homologue (Fig 3). The LOH analysis for mouse chromosome 8 in all mutants examined revealed six distinct patterns representing different types of mutational events (Fig 3B): 1) intragenic events (point mutations and epigenetic silencing), 2) apparent mitotic recombination, 3) deletion of the *Aprt* locus only, as defined by LOH restricted to the *Aprt* microsatellite, 4) multilocus deletion including *Aprt* and at least one linked microsatellite repeat locus, 5) chromosome loss, and 6) discontinuous LOH. The discontinuous LOH pattern is defined as a LOH tract that is not physically linked to the mutation leading to loss of *Aprt* expression. We use the qualifier “apparent” for the mitotic recombination LOH patterns because prior studies have shown that a substantial fraction of these patterns after charged particle exposure are due to non-reciprocal chromosome translocations [14, 27] (also, see below).

The distribution of spontaneous mutations shown in Table 2 was generated as part of our previous charged particle studies (N = 149) [27, 31] and is essentially the same as that observed in a larger set of 434 spontaneous mutants examined previously [26]. Approximately half of all spontaneous mutations are due to chromosome loss (53.7%), with LOH patterns consistent with intragenic events (23.5%) and true mitotic recombination (16.8%) as the other two major categories. The classification of spontaneous mitotic recombination events was confirmed by a cytogenetic analysis in our prior study [14] and in a study by others with mouse ear fibroblasts [32].

**Table 2:** Analysis of mutant spectra for mutants isolated from ^56^Fe ion and proton irradiated kidneys^a^.

Fe ion Dose, Gy		IE	MR	CL	D = 1	D >1	DLOH	Total^b^	RS
0	Number	35	25	80	5	1	3	149	4
	%	23.5	16.8	53.7	3.3	0.7	2.0	100	5.7
									
0.25	Number	10	13	23	1	0	0	47	0
	%	21.3	27.7	48.9	2.1	0	0	100	0
	p value^c^	0.753	0.103	0.569	0.673	0.458*	0.198*	0.677	0.136*
									
0.5	Number	13	20	25	3	7	1	69	8
	%	18.8	29.0	36.2	4.3	10.1	1.4	100	11.6
	p value	0.442	**0.040**	**0.017**	0.718	**0.009**	0.774	**0.002**	**0.014**
									
1.0	Number	5	36	22	1	2	6	72	8
	%	6.9	50.0	30.6	1.4	2.8	8.3	100	11.1
	p value	**0.005**	**<0.001**	**0.001**	414	0.242	**0.040**	**<0.001**	**0.017**
									
2.0	Number	32	64	92	2	6	28	224	6
	%	14.3	28.6	41.1	0.9	2.7	12.5	100	15.2
	p value	**0.025**	**0.010**	**0.017**	0.110	0.691	**0.002**	**<0.001**	**0.001**
									
	p overall^d^	**0.015**	**<0.001**	**0.008**	0.344	**0.006**	**<0.001**		**<0.001**
	p trend^d^	**0.019**	0.053	**0.048**	0.069	0.691	**<0.001**		**<0.001**
**Proton Dose, Gy**									
0.5	Number	5	3	14	0	0	3	25	3
	%	20.0	12.0	56.0	0	0	12.0	100	12.0
	p value							0.267	**0.005**^+^
									
1.0	Number	12	8	14	0	1	5	39	6
	%	30.7	20.5	35.9	0	10.1	12.8	100	15.4
	p value							**0.021**	**0.005**^+^

^a^ Based on LOH pattern (see Fig 1 and text), mutations were classified as 1) intragenic events (IE), 2) apparent mitotic recombination (MR), 3) chromosome loss (CL), 4) deletion of the *Aprt* locus only (D = 1), 5) multilocus deletion (D>1), and 6) discontinuous LOH (DLOH). Radiation signature mutations were obtained by adding D>1 and DLOH columns. For each mutation by dose category, the number and percentage of mutant cells exhibiting a particular mutation is given, as is the p value relative to the sham control.

^b^ The number of mutant cells examined for each dose.

^c^ P values <0.05 were considered statistically significant. Significant values are highlighted in bold.

^d^ P overall is the p value for all three doses combined relative to sham control. P trend looks for a trend in the change in percentage for a given mutation as a function of dose.

* Entries marked with an asterisk have an estimated OR (and 95% CI) computed using Parzen’s median unbiased estimator.

^+^ Numbers for RS mutations were combined from 0.5 and 1.0 protons doses to determine if a significant change occurred.

We compared the distribution of mutations for *Aprt* mutant cells isolated from mice exposed to the four dose of ^56^Fe ions with the spontaneous mutation group and found significant differences for the 3 higher doses (0.5 Gy, p = 0.002; 1.0 and 2.0 Gy, p<0.001). Most of the data from the 2 Gy group was from a prior study [31], with the addition of results from 27 newly collected mutants added here. No difference was observed for the 0.25 Gy group relative to the spontaneous spectrum (p = 0.677)(Table 2). We next performed statistical analyses for the individual classes of mutations at each dose tested. Because four ^56^Fe doses were considered, we also determined whether exposure itself was correlated with a significant change (p overall) and whether a trend as a function of dose could be discerned (p trend). Clear evidence for the mutagenic effect of ^56^Fe ion exposure can be seen from the p overall values, which are changed significantly for five of the six described mutational patterns. The only exception was for single locus deletions (p = 0.344).

**Fig 4:**
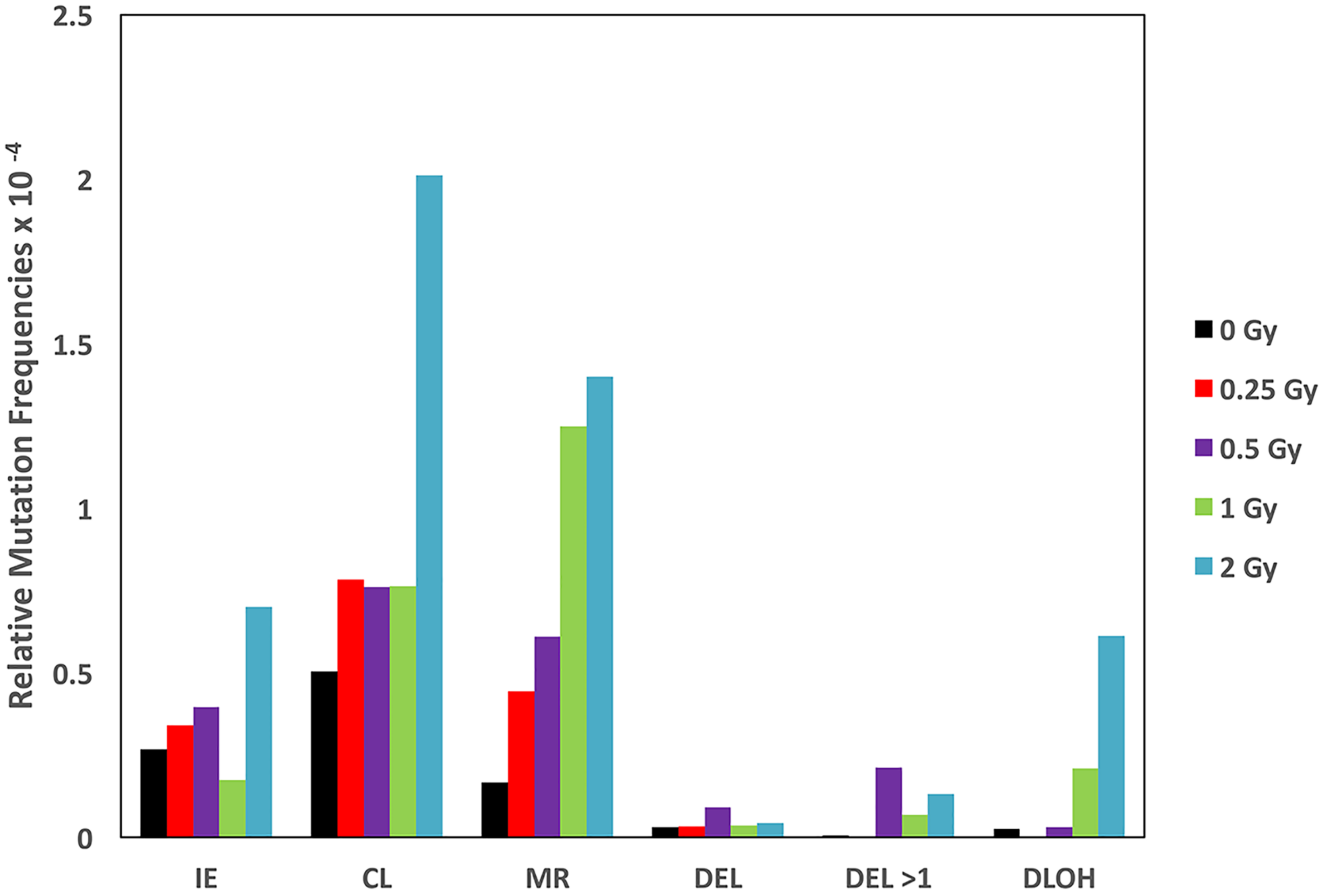
Estimated frequencies for the different mutational events. Relative mutation frequencies for different mutational events, with the overall spontaneous mutant frequency set at 1 X 10^−4^, at the different Fe ion doses were multiplied by the relative percentages of each mutational event, as defined by the LOH analysis (see Table 2), to estimate the mutation frequencies for each event. Intragenic events (IE), chromosome loss (CL), mitotic recombination (MR), interstitial deletion of *Aprt* polymorphic locus only (DEL), multilocus deletion (DEL > 1), and discontinuous LOH (DLOH). See Fig 3B for representative LOH patterns for each category.

Increased levels of apparent mitotic recombination events were observed at all four doses, with significant increases observed for the 0.5, 1.0, and 2.0 Gy doses. This increase was reflected by a p overall value of <0.001. In contrast, the percentages of the other two common mutational patterns, intragenic events (p overall = 0.015), and chromosome loss (p overall = 0.008) were decreased relative to the spontaneous mutation spectrum. However, the absolute numbers of these mutational events was apparently increased in most cases, as shown when the mutation spectra were multiplied by the mutant frequencies (Fig 4). In prior kidney mutagenesis work with high doses of γ-irradiation (7.5 Gy) [23] and Fe ions (2 Gy) [31], we showed that the least common types of spontaneous mutations, discontinuous LOH and large interstitial deletions, provide “radiation signature” (RS) mutations because they are increased as a result of exposure. Based on this earlier observation, we combined these two mutations to create a RS category for the present study. The p overall and p trend for the RS mutations were both highly significant (p<0.001) demonstrating a clear mutagenic effect, with the exception of the lowest dose tested (0.25 Gy) where no RS mutations were observed.

We also determined the mutation spectra for *Aprt* mutants isolated from kidneys exposed to 0.5 and 1.0 Gy protons (Table 2). A change relative to the 0 dose was observed for the 1.0 Gy group (p = 0.021), but not for the 0.5 Gy group (p = .267). The increase for RS mutations for the combined 0.5 and 1.0 Gy groups was statistically significant (p = 0.005).

### Translocations affecting chromosome 8 are common in ^56^Fe ion-induced Aprt mutant cells with apparent mitotic recombination LOH patterns

We showed in recent studies that a significant fraction of *Aprt* mutants induced by charged particles (5 Gy of protons or 0.2, 0.4, or 1.4 Gy Ti ions) exhibiting the “apparent” mitotic recombination LOH patterns were due to non-reciprocal chromosome translocations involving chromosome 8 [14, 27]. Here we examined a subset of *Aprt* mutants from Fe ion-exposed kidneys (0.25–1.0 Gy) exhibiting the mitotic recombination pattern with fluorescent *in situ* hybridization (FISH). Table 3 shows the number of mutant clones identified with translocations. When considering all of the ^56^Fe ion exposure mutants relative to the spontaneous mutants, a significant increase in translocations was observed as a function of dose (p = .003), which demonstrates that the ^56^Fe ions were effective at inducing translocations involved with loss of Aprt expression. We therefore consider translocations as a third type of RS event and used these fractions at each dose to estimate the number of translocations in the apparent mitotic recombination group. Using this calculation we can estimate the relative fractions of total RS mutations (translocations, large deletions, and discontinuous LOH) at each dose: 4.0% for unexposed, 12.8% at 0.25 Gy, 33.3% at 0.5 Gy and 52.8% at 1.0 Gy. We did not perform a statistical analysis for these numbers because they are estimates, although a dose response is evident.

**Table 3:** Translocations involving chromosome 8 in Fe ion-induced mutants with the mitotic recombination LOH pattern^a^.

0 Gy	0.25 Gy	0.5 Gy	1.0 Gy
1/13	3/7	6/8	5/6
7.8%	42.9%	75.0%	83.3%

^a^ The number of *Aprt* mutant clones identified with a translocation involving chromosome 8 divided by the number of mutants screened.

#### LOH events on non-selected chromosomes in Aprt mutant cells

**Fig 5:**
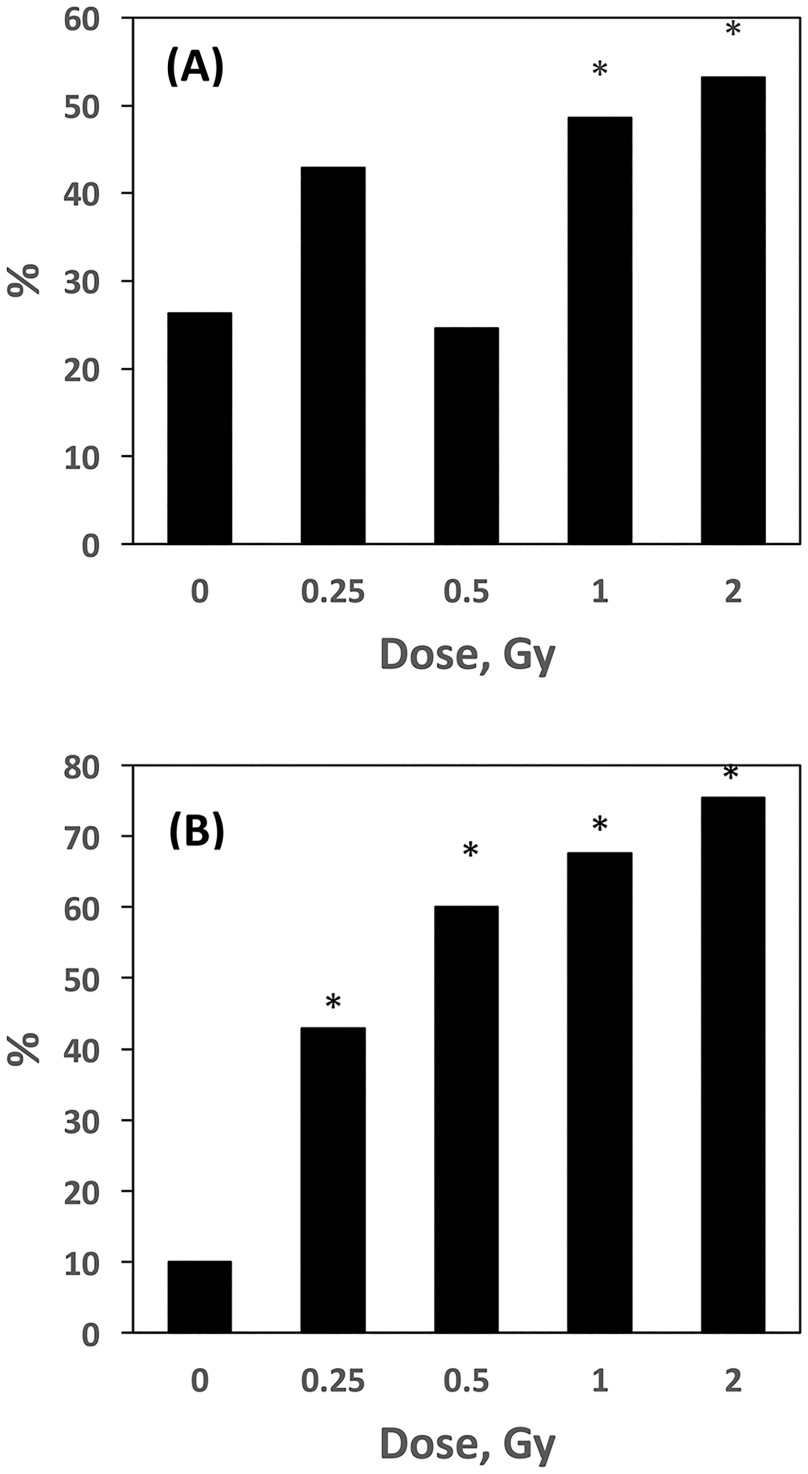
Genomic LOH in Fe ion-induced mutants. **A.** Percentage of *Aprt* mutants exhibiting genomic LOH at each dose tested. See text for more details. **B.** Percentage of *Aprt* mutants exhibiting genomic LOH that did not lose *Aprt* expression via chromosome loss. See text for more details. Asterisk (*) indicates significant difference relative to control (p<0.05). See Table 4.

In prior work with protons (4 and 5 Gy) and Ti ions (0.2, 0.4, and 1.4 Gy), we showed that *Aprt* mutant cells isolated from irradiated mouse kidneys display LOH events on other chromosomes at higher frequencies than observed in spontaneous mutants [14, 29]. We use the term “genomic LOH” to describe these mutants. Here we examined genomic LOH in the clonally derived *Aprt* kidney mutants isolated from the mice exposed to 0.25, 0.5, 1.0, or 2.0 Gy Fe ions using a set of polymorphic loci from 11 chromosome pairs [33]. When we scored the percentage of all *Aprt* mutants isolated from Fe ion exposed kidneys (i.e., pooling data from mutants isolated at all four doses) that exhibit LOH for one or more of these polymorphic loci for the exposed groups relative to the spontaneous mutants, a significant difference was observed (p = 0.001)(Fig 5A) (Table 4), though individual analyses showed significant differences only for the 1.0 (p = .009) and 2.0 Gy (p < .001) samples. An analysis for a positive linear trend was not significant (p = 0.09), due primarily to the 0.5 Gy data. We also examined 43 non-mutant clones from 2.0 Gy Fe ion-exposed kidneys and found no instances of LOH for the 11 scanned chromosomes or for chromosome 8, which demonstrates that genomic LOH is not simply due to radiation exposure. A significant increase in genomic LOH was also observed for the *Aprt* mutants isolated from kidneys exposed to 0.5 Gy protons (56.0%, p = 0.025) and a marginal difference for 1.0 Gy protons (45.0%, p = 0.065) (Table 4). The combined proton data has a p value of 0.002. Data from a prior study [29] shows that these percentages are not markedly different at higher proton doses (45.8% at 4 Gy and 63.3% at 5 Gy).

**Table 4:** Analysis of genomic LOH in mutants from ^56^Fe ion and proton irradiated kidneys^a^.

Fe ion Dose, Gy		Total^c^	Clones w/ LOH^d^	Non-CL^e^	LOH Ch14^f^
0^b^	Number	114	30	3	5
	%		26.3	10.0	4.4
					
0.25	Number	49	21	9	13
	%		42.9	42.9	26.5
	p value^g^		0.159	**0.026**	**0.001**
					
0.5	Number	61	15	9	7
	%		24.6	60.0	10.4
	p value		1.000	**0.001**	0.317
					
1.0	Number	70	34	23	22
	%		48.6	67.6	31.4
	p value		**0.009**	**<0.001**	**<0.001**
					
2.0	Number	92	49	37	24
	%		53.2	75.5	26.1
	p value		**<0.001**	**<0.001**	**<0.001**
					
	p overall^h^		**0.001**	**<0.001**	
	p trend^h^		0.09	**0.018**	
**Proton Dose, Gy**					
0.5	Number	25	14	6	6
	%		56.0	42.9	24.0
	p value^d^		**0.025**	**0.025**	**0.003**
					
1.0	Number	40	18	9	11
	%		45.0	50.0	27.5
	p value^d^		0.065	**0.005**	**<0.001**

^a^ Genomic LOH was defined by LOH for one or more of 11 non-selected chromosomes in *Aprt* mutants. See text for more details

^b^ The spontaneous samples were isolated in two prior studies of the effects of high dose Fe ions and protons [27, 31].

^c^ The number of mutant cells examined for each dose.

^d^ The number and percentage of *Aprt* mutants exhibiting genomic LOH.

^e^ The number and percentage of Aprt mutants exhibiting genomic LOH that did not lose *Aprt* expression via loss of chromosome 8 (Non-CL) relative to number of mutants exhibiting genomic LOH (Clones w/LOH).

^f^ The number and percentage of *Aprt* mutants exhibiting LOH for chromosome 14.

^g^ P values <0.05 were considered statistically significant. Significant values are highlighted in bold.

^h^ P overall is the p value for all three Fe-ion doses combined relative to sham control. P trend looks for a trend in the change in percentage for a given mutation as a function of dose.

In previous work with spontaneous mutants, we showed that loss of chromosome 8 in an un-irradiated tissue is predictive of, and presumably mechanistically linked to, loss of other chromosomes [33]. Thus, the vast majority of spontaneous *Aprt* mutants exhibiting genomic LOH are in the subset that also lost chromosome 8. As shown in Fig 5B and Table 4, only 10% (3 of 30) of spontaneous *Aprt* mutants with genomic LOH were due to other types of events on chromosome 8 (two apparent MR events and one IE event). In contrast, the frequencies of *Aprt* mutant cells exhibiting both genomic LOH and mutational events other than loss of chromosome 8 increased as a function of ^56^Fe ion dose from 42.9% (0.25 Gy) to 75.5% (2.0 Gy) for *Aprt* mutants from exposed kidneys (Fig 5B). The results for each dose were statistically significant (Table 4) and a positive linear trend with dose was observed (p = 0.018). Thus, the events driving the formation of genomic LOH patterns in the Fe ion-induced mutants are distinct from those driving the formation of genomic LOH patterns in spontaneous mutants.

We also examined the linkage between genomic LOH with the underlying events driving mutation at the *Aprt* locus in proton mutants. The results for the proton mutants were more similar to the Fe ion mutants than to the spontaneous mutants. Even at the lower proton doses used in the present study, the chromosome 8 events most closely associated with genomic LOH occurred via mechanisms other than loss of chromosome 8, though the number of mutants available at each dose (0.5 and 1.0 Gy) were less than those from the Fe ion samples (Table 4). We did not examine these results for a linear trend because only two doses were tested.

**Fig 6:**
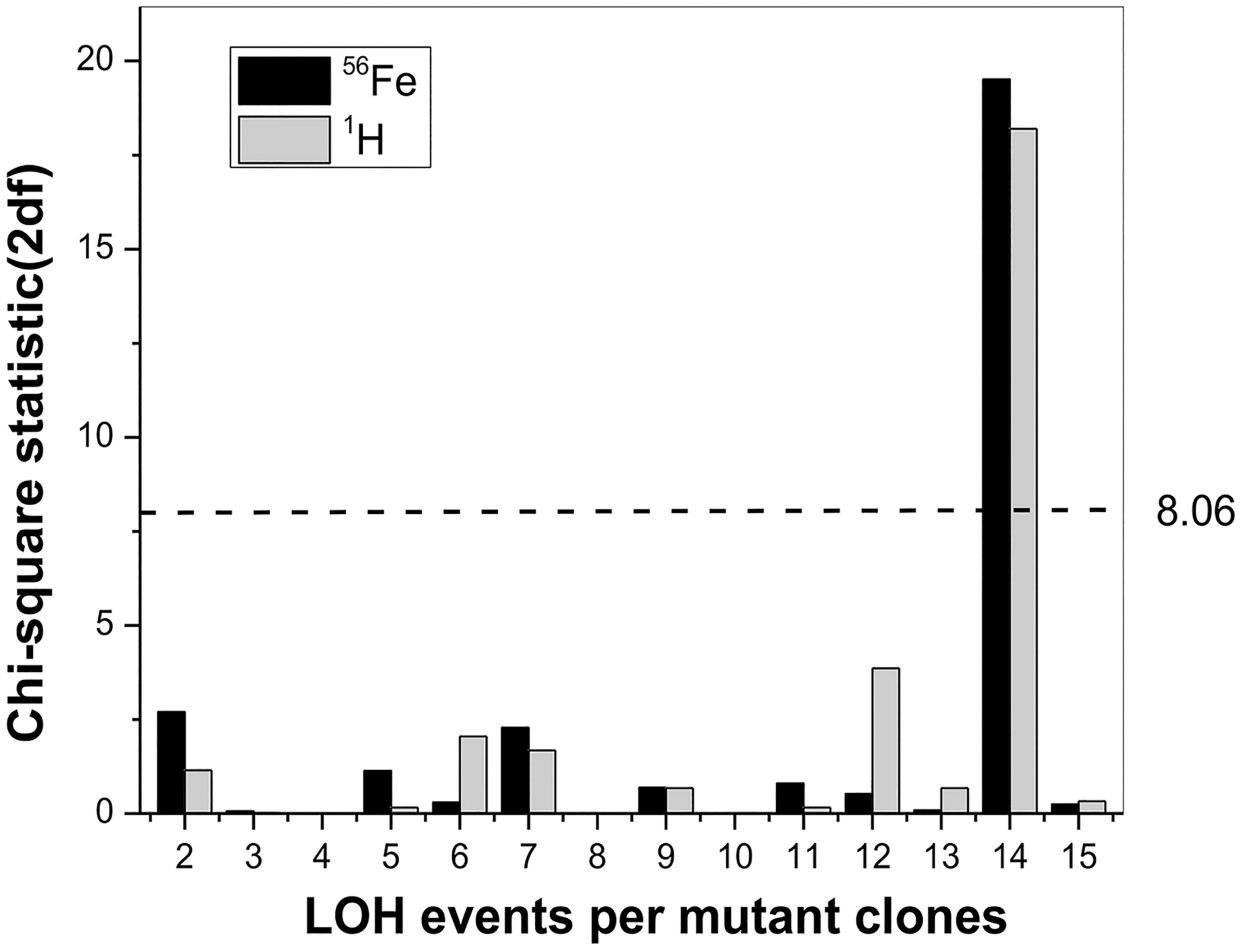
Distribution of LOH events on non-selected chromosomes in the *Aprt* mutants from Fe ion and proton-exposed kidneys. A Chi-square analysis was used to assess the distribution of non-selected chromosomes LOH events in *Aprt* mutants from Fe ion and proton-exposed kidneys. A value of 8.06 or more (dotted line) is required to conclude a chromosome has been differentially affected based on a Bonferroni adjustment. Only chromosome 14 met this threshold.

Our prior work also showed a bias for LOH events on chromosome 14 in mutants isolated from high doses of protons or Ti ions [14, 29]. A chi-square analysis was used to assess the distribution of LOH events on non-selected chromosomes for the Fe ion and low dose proton-induced mutants in the present study. We observed the same bias for changes involving chromosome 14 for the *Aprt* mutants isolated from the Fe ion and proton exposed kidneys, which requires a chi-square statistic for changes on any given chromosome must exceed a value of 8.06 to reach significance (Fig 6 and Table 4).

### SNP analysis for genomic LOH mutants

**Fig 7:**
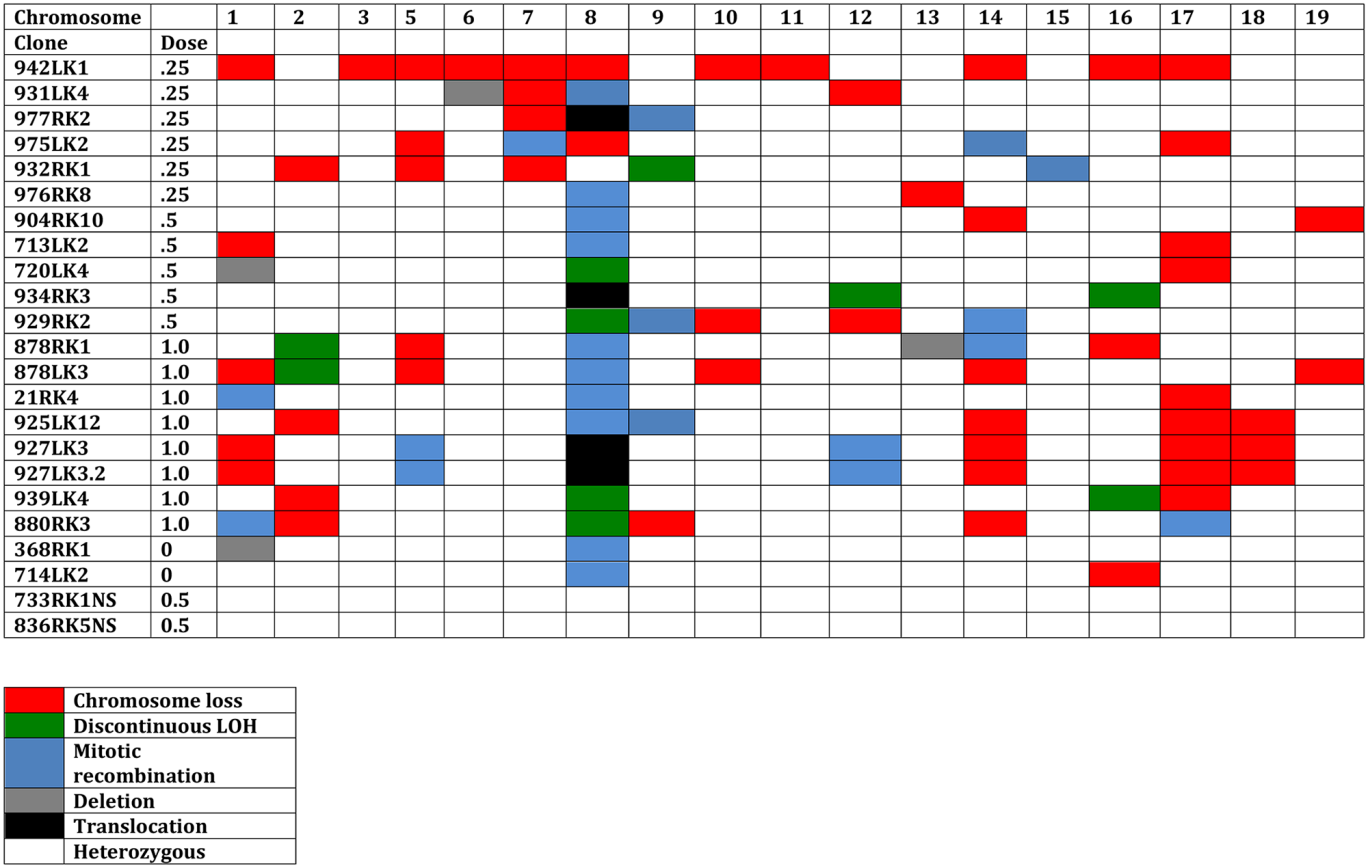
Genomic SNP analysis for *Aprt* mutants from Fe ion exposed kidneys. DNA from each mutant shown was analyzed for loss or retention of heterozygosity for multiple SNPs along each chromosome and the molecular patterns used to identify LOH tracts on affected chromosomes and used to determine the type of mutation. A color key is provided at the bottom left of the figure. The *Aprt* mutants were selected for different types of mutations affecting chromosome 8 and a subset were identified with FISH analysis for translocations (marked with black). Mutant 932RK1 has an intragenic mutation affecting *Aprt* expression. This figure also shows results for two spontaneous *Aprt* mutants (0 Gy) and two non-selected clones from kidneys exposed to 0.5 Gy. Chromosome 4 is omitted from this figure (see text).

To obtain an in-depth picture of the extent of genomic LOH in Fe ion-induced mutants, and the types of genomic change that occur, we used a SNP analysis to examine *Aprt* mutants from exposed kidneys in 18 mutants (Fig 7). This analysis is possible due to genetic diversity between the C57BL/6 and DBA/2 strains that were used to make the B6D2F1 hybrids for this study. The mutants were chosen based on a mix of mutational patterns on chromosome 8 which led to loss of *Aprt* expression. The analysis included mutants from kidneys exposed to 0.25 Gy (N = 6), 0.5 Gy (N = 5), and 1.0 Gy of Fe ions (N = 7). We ran one DNA sample twice (927LK3 and 927LK3.2, 1.0 Gy) to assess the reproducibility of the technique, which was confirmed because alterations affecting eight different chromosomes were observed and were identical in the two samples (see Fig 7). We also examined 2 spontaneous mutants with chromosome 8 “apparent” mitotic recombination patterns and 2 non-selected clones from mouse kidneys exposed to 0.5 Gy ^56^Fe ions.

The data from the SNP analysis broadened the view of the genome by including all of the chromosomes and confirmed that the genomes of *Aprt* mutants isolated from the Fe ion irradiated kidneys can carry significant mutational loads. Nine mutants from irradiated kidneys contained 5 or more affected chromosomes (excluding chromosome 4, see below), including one (942LK2) that showed loss of 11 of 18 autosomes (again excluding chromosome 4). The average number of affected chromosomes per mutant from the Fe ion exposed kidneys was 4.8. For the 2 spontaneous mutants, one lost chromosome 16 and the other had a relatively small interstitial deletion on chromosome 1, in addition to mitotic recombination for chromosome 8 in both mutants. No affected chromosomes were observed in the two non-selected clones from kidneys exposed to 0.5 Gy, consistent with the lack of change observed with the PCR-based LOH analysis of 43 non-selected clones from kidneys exposed to Fe ions.

Chromosome 4 was affected in 16 of 18 Fe ion mutants examined. Both spontaneous mutants also lost chromosome 4. Thus, changes affecting chromosome 4 appear to be a non-specific effect, and as noted by others is a common occurrence for mouse kidney cells in cell culture conditions [34]. Therefore, results for this chromosome were omitted from Fig 7.

## Discussion

Our *in vivo* mutagenesis system allows radiation-induced mutations to develop in the normal tissue environment, as would occur in the bodies of astronauts exposed to the space radiation environment to increase their risk for cancer. *Aprt* mutant frequencies from the Fe ion-exposed kidneys showed a linear increase with dose. This result is consistent with, and extends to lower doses, a prior study of Fe ion mutagenesis with doses ranging from 1.0 to 2.0 Gy [12]. In contrast, no significant increase in *Aprt* mutant frequencies was observed with the proton doses tested here (0.5 and 1.0 Gy), consistent with a previously reported lack of an increase at 3.0 Gy and an exponential increase at 4.0 Gy and 5.0 Gy [13]. The combined results for proton mutagenesis demonstrate a linear-quadratic mutagenic effect from these sparsely ionizing protons, which is consistent with the results of a study with B6D2F1 *Aprt* heterozygous kidney cells exposed in culture to the same proton beam [13]. In a prior study with Ti ions (0.2 to 1.4 Gy)[14] we observed a linear increase at the lowest doses and then a plateau in mutant frequency (i.e., a bending of the curve [35]). Thus, our results with three different charged particles demonstrate radiation quality effects for the induction of mutations in an epithelial cell type when a range of doses is considered. Moreover, the linear increases in mutant frequencies at lower doses for the high LET Fe and Ti ions suggest straightforward mutagenic mechanisms involving nuclear traversals leading to DNA damage and mutations.

The protons used in the present study are sparsely ionizing, in contrast to the densely ionizing Fe ions. The lack of a statistically significant increase in *Aprt* mutant frequencies at 0.5 and 1.0 Gy protons, despite clear mutagenic effects at higher doses, suggests a greater ability for repair of proton-induced DNA damage at these doses compared with high LET Fe ions. Indeed, it takes ~ 630 protons to deliver the same total dose as a single Fe ion and the spatial distribution of the damage is different [20, 21]. However, as discussed below, the situation is more complicated when mutational events are examined.

Based on prior studies with high dose ^137^Cs-gamma radiation [23], protons [27], and Fe ions [31], we proposed LOH patterns consistent with large interstitial deletions and discontinuous LOH as radiation signature mutations for the mouse kidney. More recently, we confirmed the usefulness of these radiation signature mutations to demonstrate mutagenic effects with lower doses of Ti ions in the kidney epithelium (0.2 and 0.4 Gy)[14], and lower doses of Ti (0.2 and 0.4 Gy) and Fe ions (0.25 and 0.5 Gy) in splenic T cells [15]. While radiation signature mutations are also observed in spontaneous mutants from unexposed tissues, they are present at statistically reduced frequencies relative to exposed tissues. Radiation signature mutations in the form of interstitial deletions have been reported recently to account for loss of the *Ptch1* locus on chromosome 13 in gamma ray-induced medulloblastomas in *Ptch1* heterozygous mice [36].

In the current study, we observed increased radiation signature mutations at 0.5, 1.0, and 2.0 Gy Fe ions. Increased interstitial deletions were observed at 0.5 Gy whereas increased discontinuous LOH was observed at 1.0 and 2.0 Gy. TThe shift in spectrum could reflect that a greater number of double strand breaks are required for the discontinuous LOH pattern to emerge. Radiation signature mutations induced by protons at the two doses tested here (0.5 and 1.0 Gy), and at higher doses tested previously (4.0 and 5.0 Gy) [27], were also predominately due to events leading to the discontinuous LOH pattern.

When considering the same doses for Fe ions and protons (0.5 and 1.0 Gy), similarities and differences are observed. At the lower dose of 0.5 Gy for protons, the mutation spectrum for chromosome 8 could not be distinguished from that for the spontaneous mutants, while the mutation spectrum for 0.5 Gy Fe ions was significantly different from the spontaneous spectrum. At the higher dose of 1.0 Gy, a statistical shift was observed for the mutational spectrum for both proton and Fe ion-induced mutants. This reflects the increased proportion of radiation signature mutations. However, the Fe ion-induced mutation group at 1.0 Gy also displayed a significant increase in apparent mitotic recombination mutants (50% of all mutants) relative to protons (20% of all mutants). As noted immediately below, this increase after Fe ion exposure reflects chromosome translocations

Chromosome aberrations are a well-defined biomarker for radiation exposure [37–39]. Although most chromosome aberrations that form initially are associated with cell death, radiation-induced translocations are often stable. We took advantage of this stability to link translocations affecting chromosome 8 to loss of a phenotype (i.e., loss of *Aprt* expression) to provide an additional signature of radiation mutagenesis [14, 27]. In this respect, *Aprt* is a particularly sensitive target for detecting stable translocations because it is located 7 Mb away from the telomere. Thus, most breaks on chromosome 8 will lead to loss of *Aprt*, and a translocation will be observed if the remaining centromeric region joins with a telomeric region of another chromosome.

We know from our prior work with 5 Gy of protons and 0.2 to 1.4 Gy of Ti ions that a significant fraction of induced mutants exhibiting the mitotic recombination LOH patterns in the kidney epithelium are actually due to translocations involving chromosome 8, as opposed to the “true” mitotic recombination events that are common in spontaneous mutants [14, 27]. Here we show increased translocation frequencies involving chromosome 8 in the Fe ion-exposed kidneys at the doses tested (0.25, 0.5, and 1.0 Gy) providing additional support for low dose and fluence effects. Overall, the translocation data support a role for multiple, double strand breaks in Fe ion-induced mutants and that somewhat surprisingly these events will frequently lead to translocations, as opposed to simple chromosome loss or interstitial deletions that do not require breaks on multiple chromosomes. As a result, stable translocations affecting chromosome 8 likely represent the largest class of radiation signature mutations in the Fe ion-induced *Aprt* mutant cells.

In prior studies, we showed that charged particle-induced *Aprt* mutant kidney epithelial cells often exhibit mutations on non-selected chromosomes (termed genomic LOH), as detected with a LOH analysis for a polymorphic marker on each of 11 additional chromosomes. The demonstration of a radiation effect, however, is complicated by the observation that genomic LOH is common in a subset of spontaneous *Aprt* kidney mutants isolated from unirradiated kidneys; specifically those that lost *Aprt* expression due to loss of chromosome 8. The proposed explanation for this class of spontaneous mutants is that loss of multiple chromosomes is common in near tetraploid cells [33], which are relatively common in the mouse kidney (~ 10% of all cells; unpublished observation). In contrast, genomic LOH is far less common in spontaneous *Aprt* mutants that lose *Aprt* expression via other mutational events on chromosome 8 (e.g., mitotic recombination and intragenic events) (See Fig 5B). We showed in this study that genomic LOH in charged particle-induced mutants is independent of the mutation leading to loss of *Aprt* expression (see Table 4 and Fig 5B) [14, 29]. We also showed that chromosome 14 is preferentially affected in *Aprt* mutants isolated at most Fe ion (except 0.5 Gy) and both proton doses tested. These results, in total, suggest strongly that genomic LOH in radiation-induced mutants is mechanistically distinct from that occurring spontaneously.

The full genomic LOH analysis using polymorphic SNPs for a subset of *Aprt* mutants from Fe ion-exposed kidneys confirmed that these cells can carry a substantial mutational load regardless of the exposure dose, and that these mutations occur via a variety of mechanisms linked to double strand breaks. This observation raises the question of whether these events occur at a single point in time as a direct result of exposure or evolve over time after exposure in the tissue environment. The latter possibility is consistent with radiation-induced genomic instability [40–42]. The former possibility of numerous changes occurring simultaneously from a single event is consistent with the hypothesis that cancers evolve rapidly in bursts of multiple genetic changes [43]. We favor the explanation that the genomic changes occur rapidly from radiation exposure due to the complexity of the initial damage associated with individual particle traversals by densely ionizing Fe ions [44, 45]. If so, a reasonable hypothesis is that when particular combinations of affected chromosomes occur within a given cell following an exposure to even a single high LET charged particle, those cells begin the malignant process. A testable prediction of this hypothesis is that charged particle-induced tumors in the kidney in a susceptible strain such as *Tsc1* or *Tsc2* heterozygous mice [46] will carry a higher mutation load than spontaneous tumors. Another testable prediction is that tumors arising after a low fluence exposure to densely ionizing heavy ions may appear earlier or be more aggressive than spontaneous tumors due to the complexity of the initial damage. This has been observed in some types of tumors induced by densely ionizing Fe ions [47, 48]. We note that mutant load in the kidney can be affected somewhat by toxicity because cells that lose mitotic capacity cannot form mutant clones (or cancer if this endpoint is studied). However, at the space relevant fluences (1 or 2 Fe ions per cell nucleus), the toxicity levels were minimal and unlikely to play a significant role in observed mutant frequencies.

The data presented here are useful for continued refinement of risk estimates because of the well-described relation between mutation and cancer [49, 50]. The deep space environment, where astronauts will spend considerable time during exploration class missions, includes heavy ions such as the Fe ions employed here. It has been estimated that 33% of the nuclei of cells in the human body will be traversed by one or more ions with Z>10 for a three-year mission in a heavily shielded configuration, e.g. to Mars, and that only about 6% would be traversed by two such ions over the duration of the mission [51]. Our results confirm and extend our findings with Ti ions [14] showing that even individual heavy ion traversals can lead to extensive genomic rearrangements in a subset of cells that survive in the normal tissue environment. The type of genomic change produced by low fluence exposure to heavy ions is reminiscent of the extensive rearrangements seen in tumor genomes.

## Conclusions

We have shown that charged particle-induced mutant cells can carry large mutational loads affecting multiple chromosomes, that not all chromosomes are affected equally, and that cells with greatly perturbed genomes remain in the intact tissue for many months after irradiation. The results indicating extensive genomic damage following low fluence exposure to densely ionizing Fe ions are of concern for realistic exposures of astronauts to the galactic cosmic radiation during extended space flight. Moreover, the results for the lower dose proton exposures are also relevant, primarily for a rare subset of solar particle events where high-energy protons may be produced at high dose-rates [52]. The linkage between these widespread genomic mutations and charged particle-induced tumors remains to be determined.
